# A case of afferent limb obstruction and gastrocolic fistula in a patient with a Billroth II gastrectomy solved by endoscopic ultrasound-guided gastroenterostomy

**DOI:** 10.1055/a-2418-0630

**Published:** 2024-10-25

**Authors:** Sifan Liu, Zheng Zhang, Ningning Dong, Wenjing Wang, Peng Li

**Affiliations:** 1 Department of Gastroenterology, Beijing Friendship Hospital, Capital Medical University, Beijing, China

Afferent limb obstruction may occur following a Billroth II gastrectomy 1 , and EUS-guided gastroenterostomy (EUS-GE) represents a novel therapeutic option for this condition 2 . In this case, we opted for this innovative treatment approach.

A 62-year-old male with a history of Billroth II gastrectomy for duodenal ulcer presented with epigastric pain and eating difficulties. The patient had a past medical history of cholelithiasis, choledocholithiasis, cholecystitis, acute pancreatitis, and pancreatic pseudocyst. Computed tomography (CT) revealed duodenal dilation and fluid accumulation ( Fig. 1 ). Gastroscopy revealed a 1.0 × 0.8-cm ulcer adjacent to the gastrojejunostomy anastomosis, with a fistula opening through which the endoscope could pass into the distal colon.

**Fig. 1:**
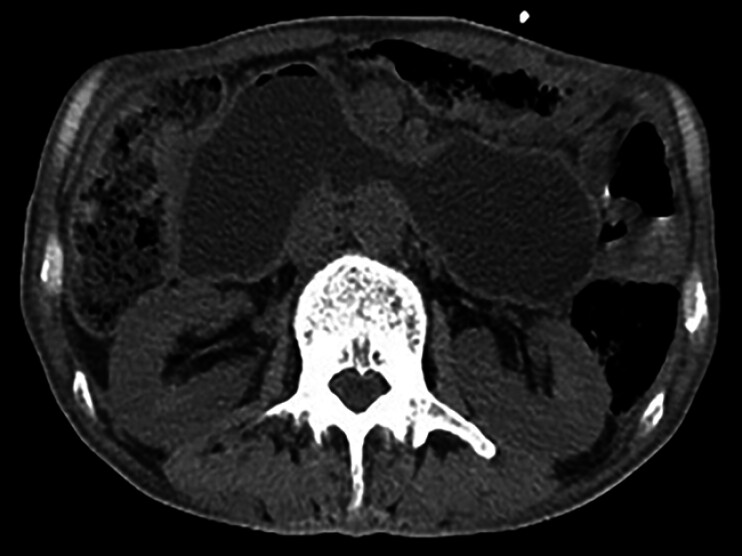
Computed tomography showed duodenal dilation and fluid accumulation.

We used argon plasma coagulation (APC) and an over-the-scope clip to close the fistula. Then we performed EUS-GE for the afferent limb obstruction ( Video 1 ). An EUS-guided 19G needle was employed for puncture, with Doppler ultrasound to prevent vascular injury and avoid other bowel segments. After the contrast media was injected to confirm bowel lumen visibility, a HOT AXIOS stent was deployed under X-ray and EUS to create a gastroenteric anastomosis and was fully expanded. The proximal end of the stent was positioned within the gastric cavity, and the lumen of the stent was patent. No adverse events occurred. On the second postoperative day, the patient was able to tolerate a liquid diet without vomiting. One week postoperatively, the gastroscopy showed the stent was in place and patent, with the proximal end in the gastric cavity and accessible to the afferent limb. Abdominal CT indicated a significant reduction in duodenal dilation and fluid accumulation ( Fig. 2 ). We postulate the patientʼs afferent limb obstruction and gastrocolic fistula may be attributed to localized inflammatory edema and pancreatic fluid accumulation related to his past medical history. The patient experienced no further eating difficulties and was discharged in improved condition.

**Fig. 2:**
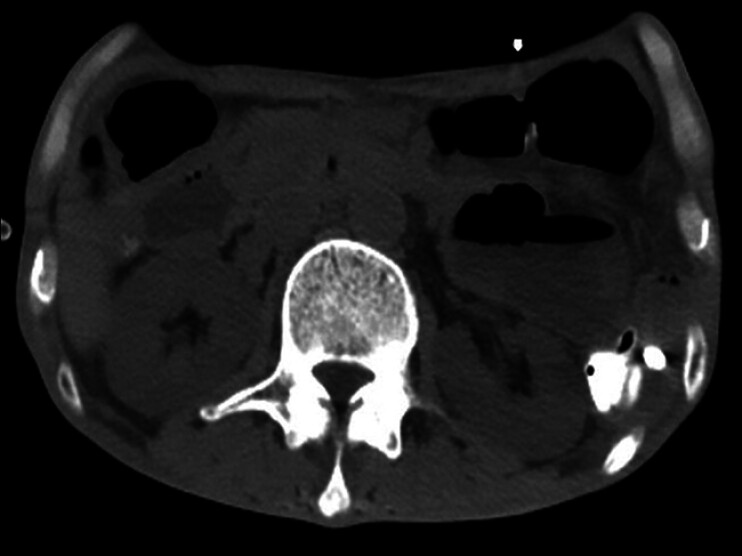
Computed tomography indicated a significant reduction in duodenal dilation and fluid accumulation.

Endoscopic ultrasound-guided gastroenterostomy to treat an afferent limb obstruction.Video 1

Endoscopy_UCTN_Code_TTT_1AS_2AC

## Notes

Endoscopy E-Videos https://eref.thieme.de/e-videos
*E-Videos* is an open access online section of the journal *Endoscopy* , reporting on interesting cases and new techniques in gastroenterological endoscopy. All papers include a high-quality video and are published with a Creative Commons CC-BY license. Endoscopy E-Videos qualify for HINARI discounts and waivers and eligibility is automatically checked during the submission process. We grant 100% waivers to articles whose corresponding authors are based in Group A countries and 50% waivers to those who are based in Group B countries as classified by Research4Life (see: https://www.research4life.org/access/eligibility/ ). This section has its own submission website at https://mc.manuscriptcentral.com/e-videos .
